# Supplementary material for: Identification of photoperiod-induced specific miRNAs in the adrenal glands of Sunite sheep (Ovis aries)
Source: Front Vet Sci. 2022 Jul 22;9:888207. doi: 10.3389/fvets.2022.888207 (PMC9354845; doi:10.3389/fvets.2022.888207)
Supplement: Supplementary file 1 [file Data_Sheet_1.ZIP › Supplementary file/miRDeep2 file.zip!/miRDeep2 file/Know_Structure/SPLP42A1/oar-mir-29a.pdf]

miRBase precursor : oar-mir-29a
Total read count : 113790
oar-miR-29a read count : 113764
remaining reads : 26

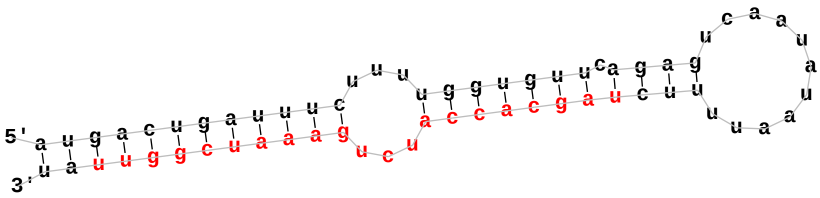

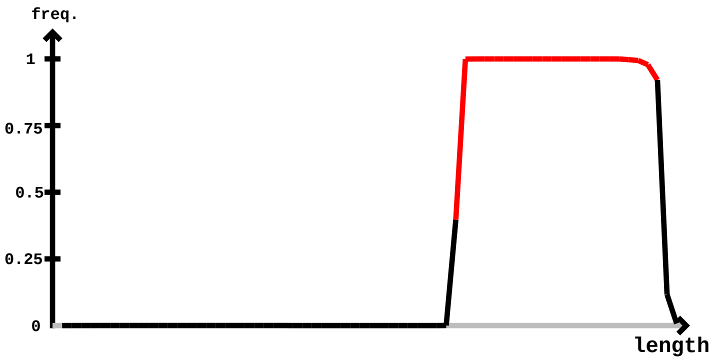

oar-miR-29a

5'- augacugauuucuuuugguguucagagucaauauaauuuucuagcaccaucugaaaucgguuau -3'	exp		
((((((((((((...(((((((.((((...........)))))))))))...))))))))))))	reads	mm	sample
.ugacugauuucuuuugguguu..	3	0	seq
.ugacugauuucuuuugguguuc...	13	0	seq
..gacugauuucuuuugguguuc...	1	0	seq
...acugauuucuuuugguguuc...	8	0	seq
......................................uucuagcaccaucugaaaucggu...	1	0	seq
....ucuagcaccaucugaaaucgg....	4	0	seq
....ucuagcaccaucugaaaucggu...	18	0	seq
....ucuagcaccaucugaaaucgguu..	5	0	seq
....ucuagcaccauUugaaaucgguu..	2	1	seq
....cuagcaccaucugaaau.......	6	0	seq
....cuagcaccaucugaaauA......	2	1	seq
....cuagcaccaucugaaauc......	66	0	seq
....cuagcaccauUugaaaucg.....	2	1	seq
....cuagcaccaucugaaaucg.....	88	0	seq
....cuagcaccaucugaaaucgg....	283	0	seq
....cuagcaccauUugaaaucgg....	1	1	seq
....cuagcaccauUugaaaucggu...	19	1	seq
....cuagcaccaucugaaaucggu...	1776	0	seq
....cuagcaccaucugaaaucgguu..	41982	0	seq
....cuagcaccauUugaaaucgguu..	183	1	seq
....cuagcaccauUugaaaucgguua.	2	1	seq
....cuagcaccaucugaaaucgguua.	693	0	seq
....cuagcaccaucugaaaucgguuau	27	0	seq
....uGgcaccaucugaaauc......	1	1	seq
....uagcaccaucugaaauU......	24	1	seq
....uagcaccaCcugaaauc......	1	1	seq
....uagcaccaucugaaGuc......	1	1	seq
....uaUcaccaucugaaauc......	3	1	seq
....uagGaccaucugaaauc......	1	1	seq
....uagcaccaucugaaauA......	4	1	seq
....uagcaccaucugaaauc......	199	0	seq
....uagcacUaucugaaaucg.....	2	1	seq
....uagcaccaucugaaaucg.....	236	0	seq
....uagcaccauUugaaaucg.....	28	1	seq
....uagcaccauUugaaaucgg....	146	1	seq

oar-miR-29a

```
augacugauuucuuuuggugucagagucaauauaauuuucuagcaccaucugaaaucgguuau
........................................uagcaccaucugaaaucgg....    1365    0    seq
........................................uagcaccauUugaaaucggu...    582     1    seq
........................................uagcaccaucugaaaucggu...    4126    0    seq
........................................uagcaccaucugaaaucgguu..    47043   0    seq
........................................uagcaccauUugaaaucgguu..    2287    1    seq
........................................uagcaccauUugaaaucgguua.    602     1    seq
........................................uagcaccaucugaaaucgguua.    11050   0    seq
........................................uagcaccauUugaaaucgguuau    44      1    seq
........................................uagcaccaucugaaaucgguuau    808     0    seq
.........................................agcaccaucugaaaucggu...    3       0    seq
.........................................agcaccaucugaaaucgguu..    34      0    seq
.........................................agcaccauUugaaaucgguua.    1       1    seq
.........................................agcaccaucugaaaucgguua.    9       0    seq
.........................................agcaccaucugaaaucgguuau    1       0    seq
..........................................gcaccaucugaaaucgguu..    3       0    seq
..........................................gcaccauUugaaaucgguu..    1       1    seq
```
